# Green synthesis of Ag nanoparticle supported on graphene oxide for efficient nitrite sensing in a water sample

**DOI:** 10.1038/s41598-023-46409-0

**Published:** 2023-11-09

**Authors:** Nourhan Ezzat, Mahmoud A. Hefnawy, Shymaa S. Medany, Rabab M. El-Sherif, Sahar A. Fadlallah

**Affiliations:** 1 Bio-Nanotechnology Department, Faculty of Nanotechnology, Cairo University, Giza, 12613 Egypt; 2 Chemistry Department, Faculty of Science, Cairo University, Giza, 12613 Egypt; 3 Biotechnology Department, Faculty of Science, Cairo University, Giza, 12613 Egypt

**Keywords:** Materials for energy and catalysis, Environmental sciences

## Abstract

Water is essential for conserving biodiversity, ecology, and human health, but because of population growth and declining clean water supplies, wastewater must be treated to meet demand. Nitrite is one of the contaminants in wastewater that is well-known. It is crucial to identify nitrite since it can be fatal to humans in excessive doses. Utilizing a straightforward and effective electrochemical sensor, nitrite in actual water samples may be determined electrochemically. The sensor is created by coating the surface of a GC electrode with a thin layer of graphene oxide (GO), followed by a coating of silver nanoparticles. The modified electrode reached a linear detection range of 1–400 µM. thus, the activity of the electrode was investigated at different pH values ranging from 4 to 10 to cover acidic to highly basic environments. However, the electrode recorded limit of detection (LOD) is equal to 0.084, 0.090, and 0.055 µM for pH 4, 7, and 10, respectively. Additionally, the electrode activity was utilized in tap water and wastewater that the LOD reported as 0.16 and 0.157 µM for tape water and wastewater, respectively.

## Introduction

Water is crucial for maintaining human health and preserving the ecosystem and biodiversity. Unfortunately, rapid population, urbanization, climate change, and pollutants have all decreased drinkable water resources. The disparity between water availability and demand is getting more significant. It is crucial to treat wastewater to resolve the supply–demand paradox and achieve sustainability in the water management system^1,2^. Nitrates, the salt or ester anion of nitrous acid, can occur naturally or artificially in groundwater. Nitrates from fertilizers are released into the environment through runoff from sewage and mineral deposits. Uncontrolled industrial effluents can sometimes cause nitrite to permeate groundwater. Nitrate may be fatal to humans, especially in large amounts. Nitrate and nitrite can enter the body directly or indirectly through the ingestion of polluted water. In the long term, the buildup of these substances in the body causes health issues such as digestive disorders, malignancies, and even death threats in youngsters^3,4^. Wastewater treatment is becoming more accessible and efficient. Several methods, including chemical, physical, and biological ones, have been recognized for wastewater treatment; some examples are ultrafiltration, adsorption, chemical precipitation, biological oxidation, Algae, activated carbon (AC), and improved oxidation processes^5,6^. Due to their quick reaction, simple operation, and compactness, electrochemical sensors are potentially useful devices for the sensitive and focused detection of analytes^7–10^. These sensors can be used for multiple ion detection, water quality monitoring of conductivity, dissolved oxygen, and pesticides because they are primarily nonspecific^11–13^.

Due to its unique properties, nanotechnology is used in water desalination as a unique approach to wastewater treatment and water scarcity challenges.

Silver (Ag) is cheaper than platinum and palladium as noble metal catalysts. Due to their antimicrobial and oligodynamic capabilities, silver nanoparticles have been proven superior to traditional water treatment techniques. Due to this substance’s distinctive electrical, optical, and catalytic capabilities at the nanoscale, targeted medication delivery, imaging, diagnostics, and detection devices have been researched and created^14,15^. Different preparation techniques show the form and size of the silver nanoparticles, which in turn define their distinctive features. Electrochemical, chemical reduction, laser ablation, and electron irradiation are a few examples of synthetic techniques^16,17^. As catalysts and disinfectants in wastewater treatment, silver nanoparticles (Ag NPs) and their nanocomposite materials have been utilized extensively^18–20^. Because of their ultra-high surface area, well-organized structure, specific surface region, and flexible surface functionalization, Attractive graphene oxide-based nanocomposites exhibit exceptional physicochemical features such as high attractiveness, variable form and size, and ease of modification or functionalization^21–24^. Graphene Oxide has high dispersibility, making it difficult to isolate it from a fluid arrangement even after contamination adsorption. To avoid the above issue, the charge of GO is the best solution; polarized GO can be easily isolated by utilizing the attractive outer field^25,26^.

Silver nanoparticles (AgNPs) have been widely employed in electrochemical sensing. Noble metal nanoparticles, on the other hand, tend to aggregate and oxidise in practical applications because of their high surface energy and small size. Due to its synergistic electrochemical features that prevent nanoparticle aggregation and increase the stability and repeatability of silver NP, graphene has been employed as a support material for the creation of metal nanoparticles^27–30^.

It was recognized that the utilization of reducing agents in the production of silver has harmful effects on both the environment and living organisms. Consequently, there has been a significant focus on environmentally friendly preparation techniques. The utilization of green plant extracts has been widely documented in preparing silver-based composites. Examples of such extracts include Aqueous Citrus Limon Zest extract, Capsicum Chinese Plant extract, *Alhagi graecorum* Leaf extract, and *Juniperus procera* extract^31–36^.

The botanical extract comprises a variety of organic compounds, including phenols, flavonoids, carotenoids, and vitamins, which function as agents for reducing greenness^37^. Green tea extract is commonly employed in metal synthesis owing to its diverse reducing and capping agents, such as enzymes, polyphenols, and amino acids^38^.

Herein, silver nanoparticles were prepared by the green method and cast on a graphene oxide support. The produced electrode surface was used as an electrochemical sensor for nitrite in water samples. The electrode activity was investigated in acidic, neutral, and basic mediums. At various pHs, the modified electrode's calibration curve was calculated. Chronoamperometry was used to determine the detection limit and linear range. The analogous circuit for nitroxide detection on the changed surface was discovered using electrochemical impedance. Cyclic voltammetry is used in the detection of nitrite. Additionally, the charge transfer resistance for various surfaces was compared. An SEM and An XRD were used to determine the morphological and characteristic properties of the electrode.

## Experimental

### Preparation of graphene oxide

Hummer’s technique was utilized to create graphene oxide^22,24,39^. Briefly, during the initial stages of preparation, 5% HCl was applied to powdered graphite flakes. Then, after being carefully added and stirred for 2 h, H_2_SO_4_ (98%) and KMnO4 were added to the solution in the ice bath. The graphite oxidation reaction was stopped by diluting a 30% H_2_O_2_ solution with pure water. Finally, the graphene oxide was recovered by filtering and repeatedly rinsed with a 10% (HCl) solution to remove extra metal and salts.

### Preparation of Silver by green approach

The green tea extract was obtained by boiling 5 g of green tea leaves in 50 mL of double distilled water for 30 min. The fluid was subsequently subjected to a chilling procedure and filtered to remove the tea leaves. The finished solution was then put into an opaque container and put in the fridge to be used later. Silver particles were synthesized by dissolving 5.8 g of hydrated silver nitrate (20 mmol) in 50 mL of distilled water, which was subsequently transferred to a 250 mL beaker. After that, a gradual addition of 100 mL of green tea was made. The solution undergoes a chromatic transition, accompanied by the emergence of a turbid, opalescent appearance. The solution was allowed to cool in the absence of light for 5 h. Subsequently, the suspension underwent centrifugation and was subjected to an ethanol wash to eliminate any remaining residues.

### Electrode preparation

**Figure 1:**
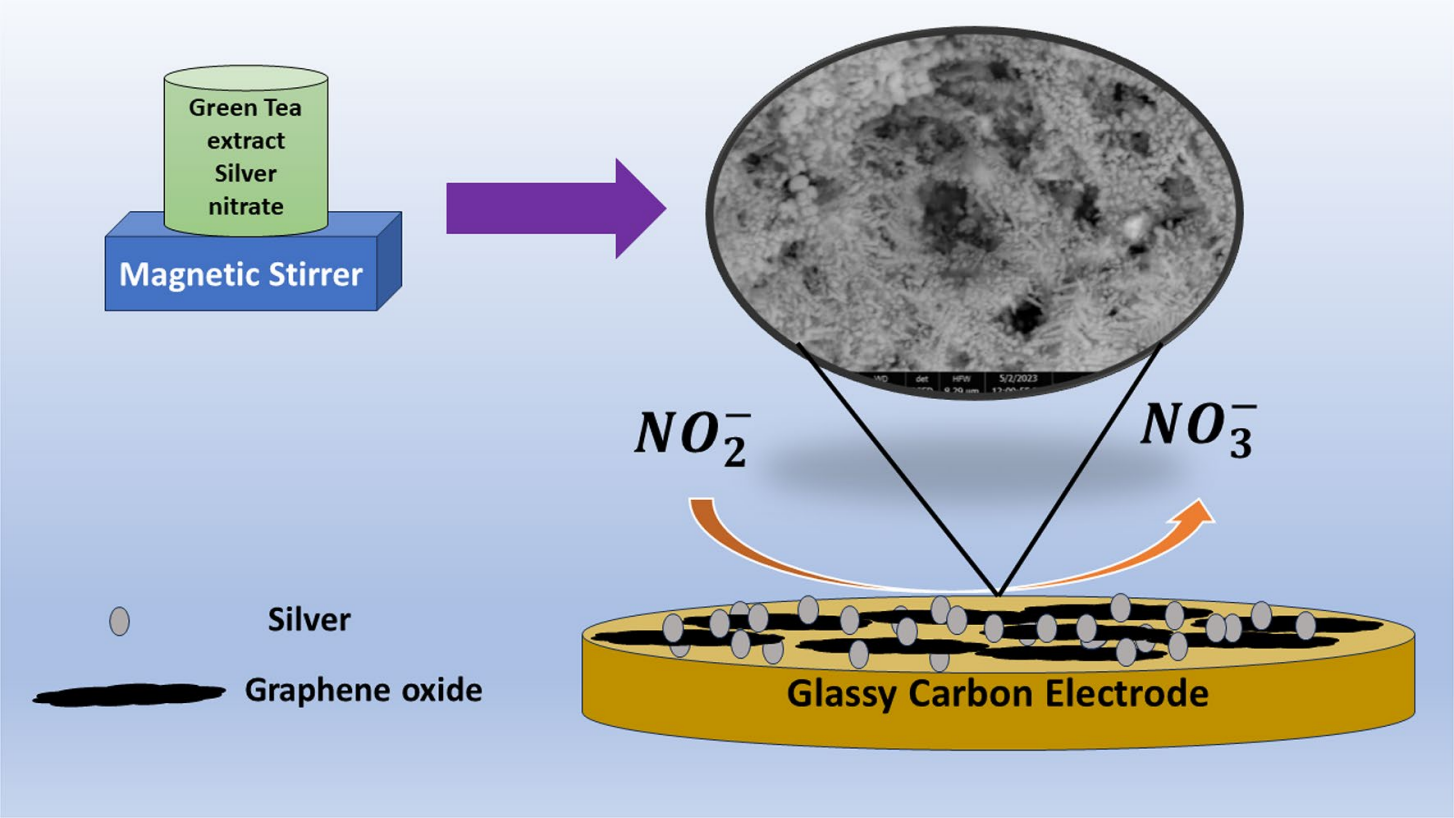
Representation of modified glassy carbon electrode with graphene oxide and silver nanoparticles.

The electrode was prepared using two suspensions: (A) containing 10 mg of silver in 1 mL of DMF, and B) containing 10 mg of graphene oxide in 1 mL of DMF. The electrode was fabricated by casting 10 µL of suspension B followed by 10 µL of suspension (A) after drying the electrode. Electrochemical measurement was examined utilizing a working electrode made of glassy carbon with a diameter of 3 mm (see Fig. 1).

Different ratios between graphene oxide and silver were selected like (1:2, 1:3, 2:1, and 3:1). The ratio of (1:1) showed the best catalytic activity and faster response time. The electrochemical measurement was conducted using the conventional three-electrode system, wherein Ag/AgCl/KCl (sat.) was employed as the reference electrode, and Pt wire was used as the auxiliary electrode. The Autolab potentiostat/galvanostat PGSTAT128N was used to measure Linear Sweep Voltammetry (LSV), Constant Potential Chronoamperometry (CA), and Electrochemical Impedance Spectroscopy (EIS). The fitted circuit was also executed using Nova (Version 2.1, Metrohm Autolab, Utrecht, Netherlands). The study involved the construction of a three-electrode cell with the electrocatalysts that were prepared. The electrodes used in the cell were the working, reference, and auxiliary electrodes made of Pt wire and Ag/AgCl/KCl (sat.), respectively. This study used the Ag/AgCl/KCl (sat.) reference electrode for all potential values. During the electrochemical impedance spectroscopy measurements, a constant AC voltage value was modified by using an AC voltage amplitude of 10 mV and a frequency range from 1 × 10^4^ to 0.1 Hz. The electrolyte utilized in the experiment was a Phosphate Buffer Solution (PBS) and was subjected to varying pH levels. The ability of the sensor was utilized in wide range of pH starting from acidic to basic range. The pH of the PBS was changed using phosphoric acid and sodium hydroxide. For real application, two different samples of water were used to investigate the activity of the electrode namely tape drinking water and wastewater.

## Result and discussion

### Structure and surface characterization

**Figure 2:**
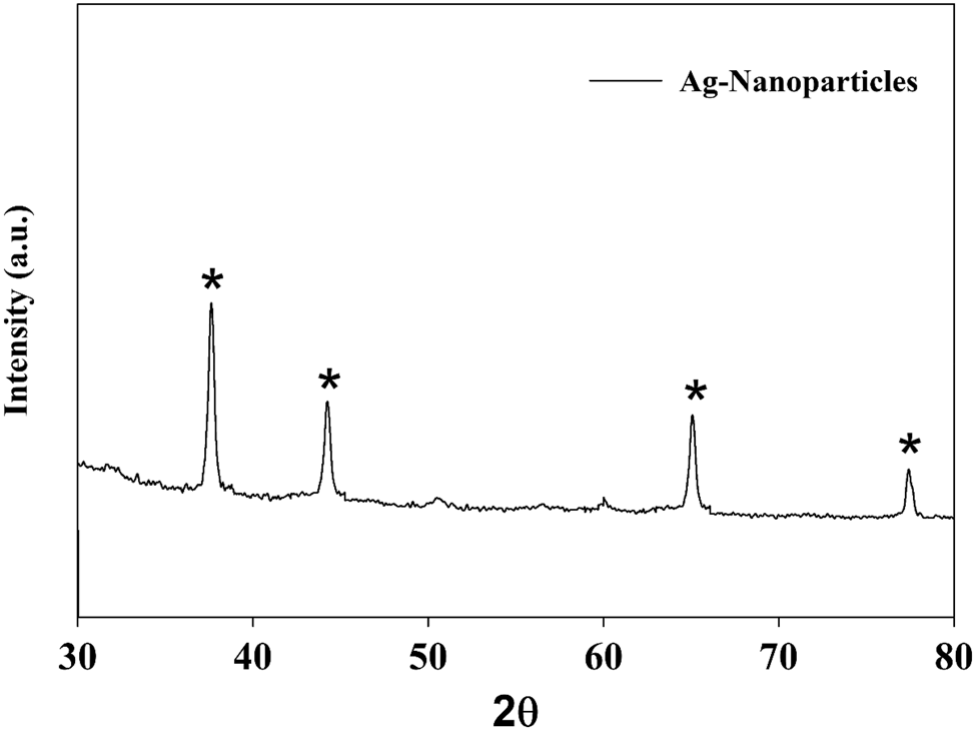
XRD chart for Ag-nanoparticles.

The chemical structure of the silver composite that was prepared underwent evaluation through the utilization of powder X-ray diffraction (XRD). Figure 2 depicts the X-ray diffraction (XRD) pattern of the GO@AgNPs composite that has been modified. As per the JCPDS reference card with file number 04-0783, it has been observed that there exist four distinct peaks at 2θ values of 37.5°, 44.3°, 64.8°, and 77.3° that values correspond to the Miller indices (111), (200), (220), and (311) respectively^40^. Based on the available data, it is probable that the structure of a silver crystal is that of a face-centred cubic (fcc) with a point group of m$\overline{3}$m.

**Figure 3:**
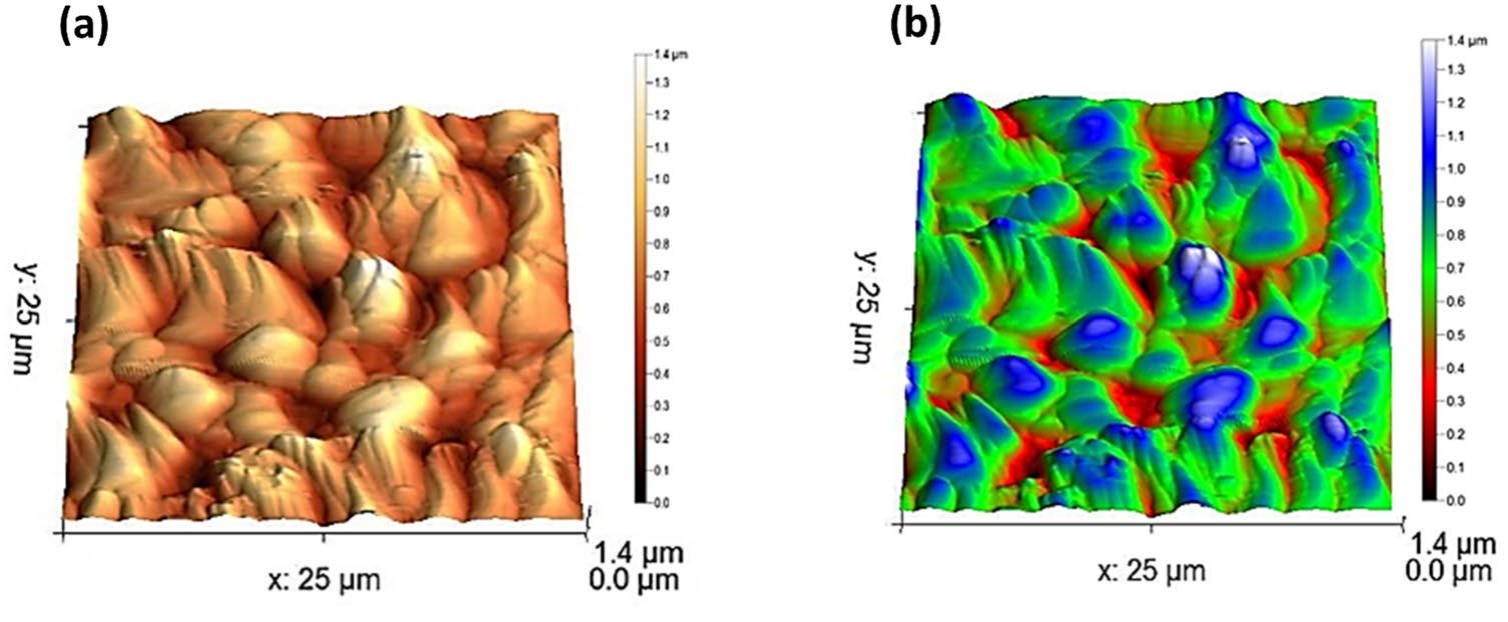
(**a,b**) AFM topography of the modified Ag/GO sample.

The surface topography of Ag@GO sample was utilized using atomic force microscopy (AFM). As represented in Fig. 3, AFM image of Ag/GO sample. Thus, expected thick graphene oxide sheets can be observed in 3D images owing to the covalently bounded oxygen and displacement of sp^3^ hybridized carbon slightly above and below carbon sheets. Furthermore, the surface roughness and surface area were evaluated using AFM. The provided surface area and mean roughness were 667.2 µm^2^, 151.6 nm respectively.

**Figure 4:**
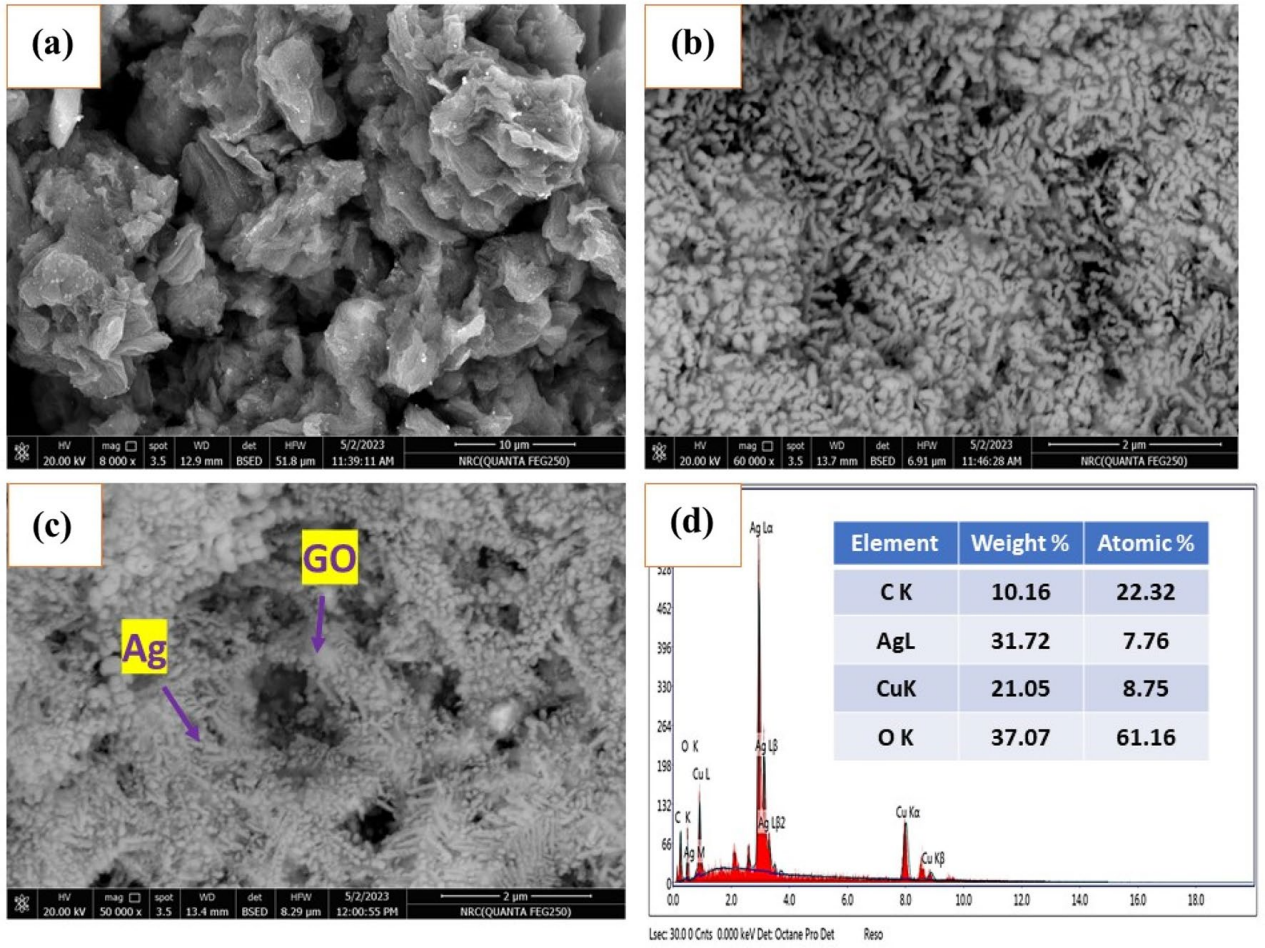
Representation of SEM of GC/GO in (**a**), the SEM of GC/Ag NPs in (**b**), the SEM of GC/GO/Ag NPs in (**c**), and the EDAX of GC/GO/Ag NPs in (**d**).

SEM is used to examine the surface morphology of the charged electrodes. The GC substrate is covered in a network of tiny filaments that resemble GO NPs on the surface (Fig. 4a). As a result, the surface area of the GC surface increases, and the conducting network is improved. A thin layer of Ag NPs is applied over the GC in Fig. 4b. The GO surface is covered in Ag NPs, as seen in Fig. 4c. More electro-active sites are available for nitrite oxidation on the resultant surface. Thus, the particle size of the silver nanoparticles was estimated to be 70–90 nm. Figure 4d depicts the GC/GO@Ag NPs surface’s EDX. The EDAX shows the components of the GO@Ag NPs composite.

### Electrochemical sensor for nitrite

**Figure 5:**
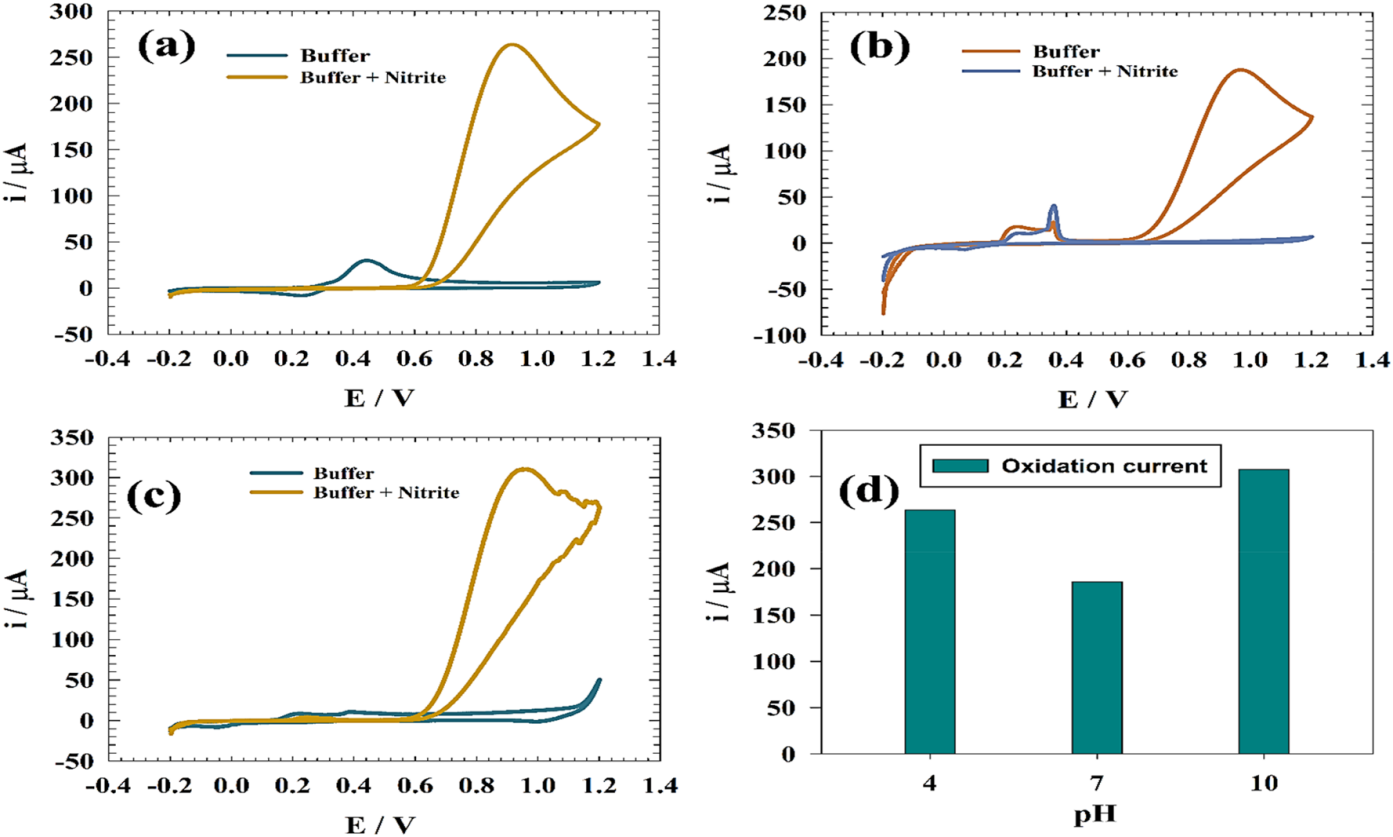
CV of the modified GC/GO/Ag in the presence and absence of nitrite at different pH mediums (**a**) Acidic, (**b**) Neutral, (**c**) Basic. (**d**) Relation between anodic current versus solution pH.

The electrochemical sensing of the nitrite was investigated using the cyclic voltammetry technique. Figure 5 shows cyclic voltammetry of GC/GO/Ag in the presence and absence of nitrite at various pH values. Figure 5a, one redox peak of silver in an acidic medium develops, the first at 0.2 V and the second at 0.4 V, as shown in Fig. 5a. The redox peak disappears when nitrite is introduced, and the nitrite oxidation peak forms at 0.94 V. In Fig. 5b, nitrite detection was investigated on an electrode in a neutral medium (pH 7) and discovered three peaks: one reduction at 0.08 V and two oxidations at 0.36 V and 0.98 V. When nitrite was added, a nitrite peak developed and proceeded to rise, and oxidation occurred. And the peak of the buffer went down. As shown in Fig. 5c, the CVs of GC/GO/Ag electrode in a basic solution (pH 10) without nitrite and discovered one reduction peak at 0.08 V and one oxidation peak at 0.2 V. When we added nitrite, the large oxidation peak reached 0.94 V. Figure 5d shows a comparison of the oxidation current of nitrite at various pH levels. Because silver changes to oxide or hydroxide, which is more persistent on the surface and has the maximum activity, oxidation of nitrite in the basic medium is greater than neutral and acidic.

### Effect of concentration

**Figure 6:**
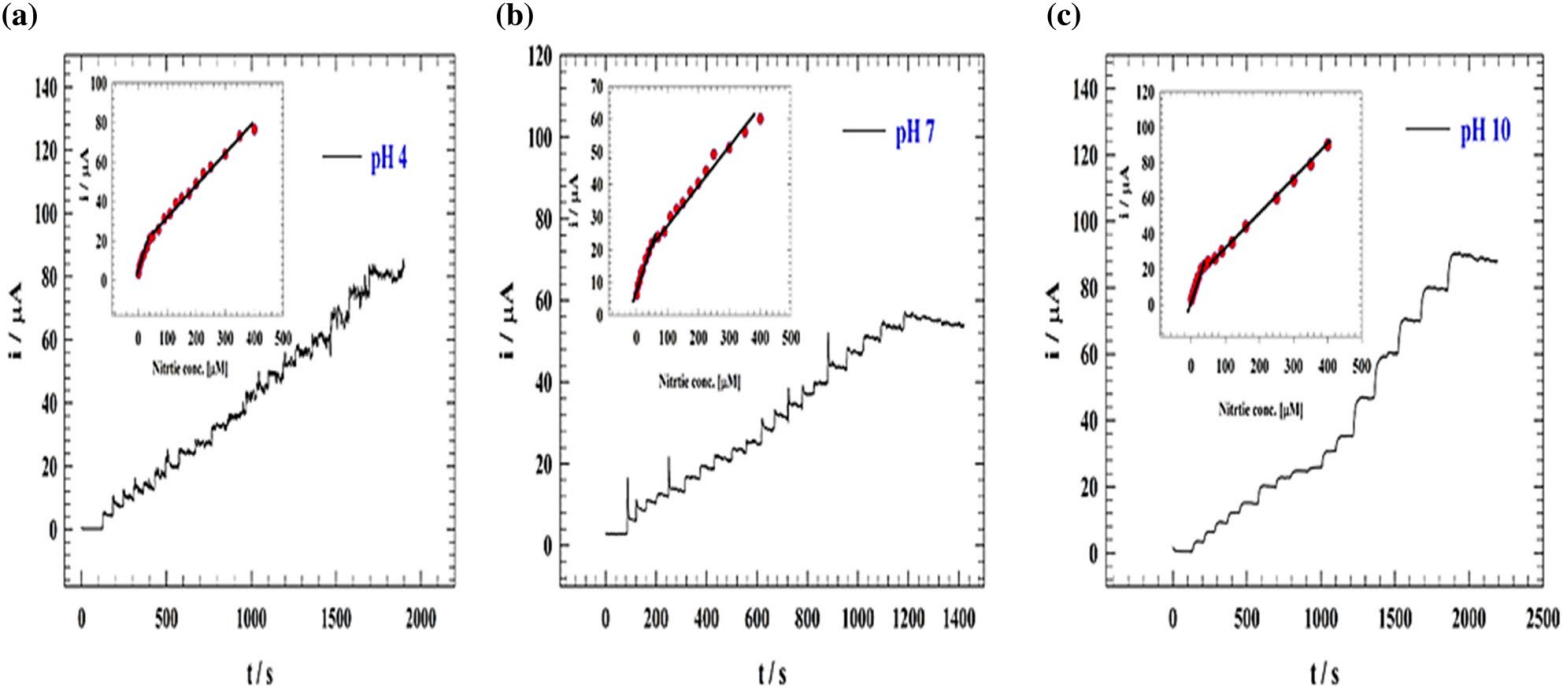
(**a–c**) Chromatograms of the modified GC/GO/Ag at different pH values. Inset figure the calibration curves.

The most typical water contaminant is nitrite. As a result, it was decided to study the calibration curve of nitrite detection and sensitivity in the concentration range that corresponded to the real pollution ranges. As a result, the Chronoamperometry (CA) approach was used at various pH levels to examine the impact of increasing the nitrite content (Fig. 6a–c). In a solution of 0.1 M PBS (pH 4, 7, 10), the calibration curve and sensitivity of the electrode were tested across the concentration range of (1 × 10^−6^ up to 400 × 10^−6^ M). Comparison between LOD and LOQ were reported for different solution pH of modified electrode in Table 1. The improved GC/GO/Ag’s Chromatograms in an acidic medium is represented in Fig. 6a. The inset figure represents the calibration curve for the modified electrode. The electrode showed two linear nitrite detection ranges for 1 to 50 µM and 50 to 400 µM. The linear relation was estimated according to the following equations:

1 $${\text{Ip }}(\upmu {\text{A}}) \, = \, 0.{3}0{\text{ C}}_{{{\text{Nitrite}}( {\upmu {\text{M}}} )}} + { 7}.{37},$$

2 $${\text{Ip }}(\upmu {\text{A}}) \, = \, 0.{\text{11 C}}_{{{\text{Nitrite}}( {\upmu {\text{M}}} )}} + { 17}.{46}.$$

**Table 1:** The values of LOD and LOQ of different media.

Media	LOQ (μM)	LOD (μM)
Acidic	0.28, 0.79	0.084, 0.23
Neutral	0.3, 0.82	0.090, 0.245
Basi	0.18, 0.47	0.055, 0.142

Otherwise, the calibration curve slope was used to estimate the limit of detection and quantization. A method for estimating the smallest quantity of analyte in a sample that can be detected is known as the limit of detection^16^. On the other hand, the lowest nitrite concentration that can be quantitatively identified using a stated accuracy and precision is known as the limit of quantization. The limits of detection (LOD) and limits of quantization (LOQ) were calculated for nitrite according to the following equations:

3 $${\text{LOD }} = {\text{ 3 s}}/{\text{m}},{\text{ and LOQ }} = { 1}0{\text{ s}}/{\text{m}},$$

where s is the standard deviation, and m is the slope of the calibration curves.

For GC/GO/Ag modified electrodes in an acidic medium, LOD were found to be 0.084 and 0.23 μM; while LOQ were 0.28 and 0.79 μM for lower and higher concentration ranges, respectively. The improved GC/GO/Ag’s Chromatograms in a neutral medium is represented by Fig. 6b. The calibration curve for the modified electrode is shown in the inset figure. The electrode showed two linear nitrite detection ranges for 1 to 50 µM and 50 to 400 µM. The linear relation was estimated according to the following equations:

4 $${\text{Ip }}( {\upmu {\text{A}}} ) \, = \, 0.{\text{32 C}}_{{{\text{Nitrite}}(\upmu {\text{M}})}} + { 7}.{158},$$

5 $${\text{Ip }}( {\upmu {\text{A}}} ) \, = \, 0.{\text{113C}}_{{{\text{Nitrite}}(\upmu {\text{M}})}} + { 17}.{15}{{.}}$$

The limits of detection (LOD) and limits of quantization (LOQ) were calculated for nitrite according to the following Eq. (3).

For GC/GO/Ag modified electrode in a neutral medium, LOD was found to be 0.090 and 0.245 μM; while LOQ were 0.3 and 0.82 μM for lower and higher concentration ranges, respectively.

The modified GC/GO/Ag Chromatograms in the basic medium is represented by Fig. 6c. The calibration curve for the modified electrode is shown in the inset figure. The electrode displayed two linear nitrite detection ranges for 50 to 400 M and 1 to 50 M, respectively. The linear relation was estimated according to the following equations:

6 $${\text{Ip }}( {\upmu {\text{A}}} ) \, = \, 0.{\text{49 C}}_{{{\text{Nitrite}}(\upmu {\text{M}})}} + { 3}.{794},$$

7 $${\text{Ip }}( {\upmu {\text{A}}} ) \, = \, 0.{\text{19 C}}_{{{\text{Nitrite}}(\upmu {\text{M}})}} + { 13}.{485}.$$

The limits of detection (LOD) and limits of quantization (LOQ) were calculated for nitrite according to the following Eq. (3).

For GC/Ag@GO modified electrodes in basic medium, LOD was found to be 0.055 and 0.142 μM. At the same time, LOQ were 0.18 and 0.47 μM for lower and higher concentration ranges, respectively Our work for nitrite determination was compared with other electrodes found in the literature and mentioned in Table 2.

**Table 2:** Comparison between different surfaces’ efficiency toward nitrite detection.

Electrode	Linear detection range (µM)	Limit of detection (µM)	Method	References
CeO_2_/La_2_O_3_	0.25 to 4000	0.015	Amperometry	^41^
Au-MoS_2_@RGO	0.2 to 2600	0.038	Amperometry	^42^
GC/PANI/NiOnF	1–500	0.064	Amperometry	^13^
MoO_3_-Co_3_O_4_	0.3125 to 4514	0.075	Amperometry	^43^
Cobalt-NFs	100 to 2150	1.2	Amperometry	^44^
Zn@Chitosan	1 to 150	0.402	Amperometry	^45^
PdO@RGO	10 to 1500	10.14	differential pulse voltammetry	^46^
GC/GO/Ag	1 to 50	0.084	Amperometry	This work
GC/GO/Ag	50–400	0.23	Amperometry	This work

### Kinetic studies

**Figure 7:**
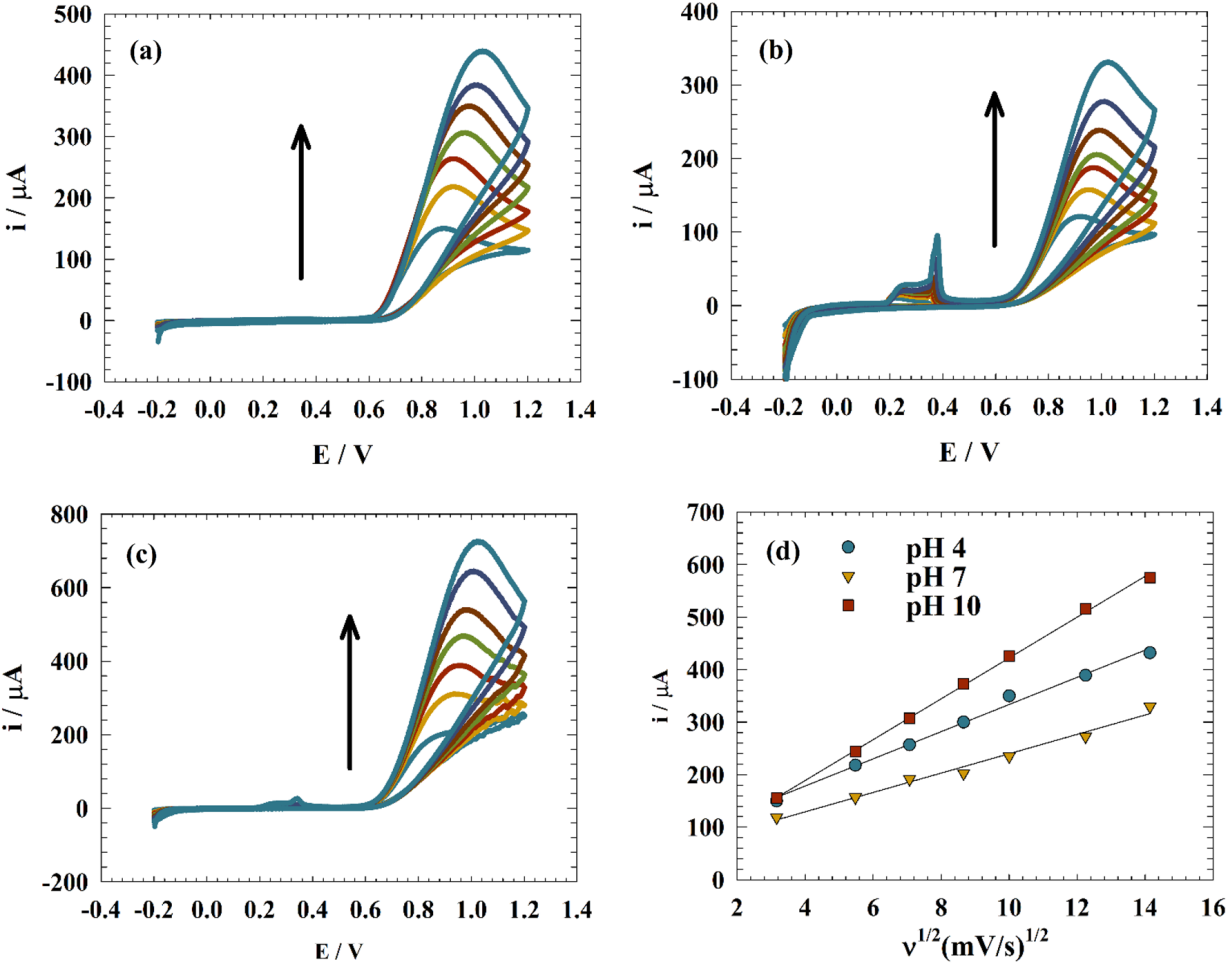
(**a–c**) CVs of modified electrode GC/GO/Ag at different pH values. (**d**) Linear relation between oxidation current versus square root of the scan rate.

Kinetic factors including diffusion and transfer coefficients, were examined to comprehend nitrite electrochemical detection on the modified electrode GC/GO/Ag. Figure 7 shows the cyclic voltammetry of the modified electrode GC/GO/Ag in a 0.1 M PBS and 0.5 mM nitrite solution at various pH levels. (i.e., 4, 7, and 10).

Figure 7a shows how the oxidation current rises as the sweep rate rises. As the scan rate increased, the peak marginally changed more positively. On the other hand, as shown in Fig. 7b, the impact of the scan rate was investigated at the neutral medium. The oxidation current of the nitrite and the silver transition (redox peak) increase when sweep rates rise. The greatest rise in the oxidation current with increasing sweep rate is seen in Fig. 7c. However, as the scan rate rose, the peak changed in a more favorable direction.

The diffusion coefficient of the nitrite was estimated using the Randles Sevcik equation^47–49^:

8 $${\text{Ip }} = \, ( {{2}.{687 } \times { 1}0^{{5}} } ){\text{ n}}^{{{3}/{2}}} \nu^{{{1}/{2}}} {\text{D}}^{{{1}/{2}}} {\text{A C}}_{{\text{o}}} ,$$

where Ip is the peak current (A), n is the number of electrons exchanged in electrochemical urea oxidation (n = 6), ν is the scan rate (V s^−1^), Co is the concentration of urea (mol cm^−3^), A is the geometrical electrode area = 0.0707 cm^2^, and D is the diffusion coefficient (cm^2^ s^−1^).

The linear relationship between the square root of the scan rate and the anodic oxidation current is seen in Fig. 7d. By measuring the slope of the linear relationship, one may determine the nitrite diffusion coefficients at various pH levels. As opposed to this, the diffusion coefficients listed for acidic, neutral, and basic media are 1.26 $\times$ ${10}^{-7}$, 2.54 $\times$ ${10}^{-7}$, and 4.87 $\times$ ${10}^{-7}$ cm^2^ s^-1^, respectively.

### Electrochemical impedance spectroscopy

**Figure 8:**
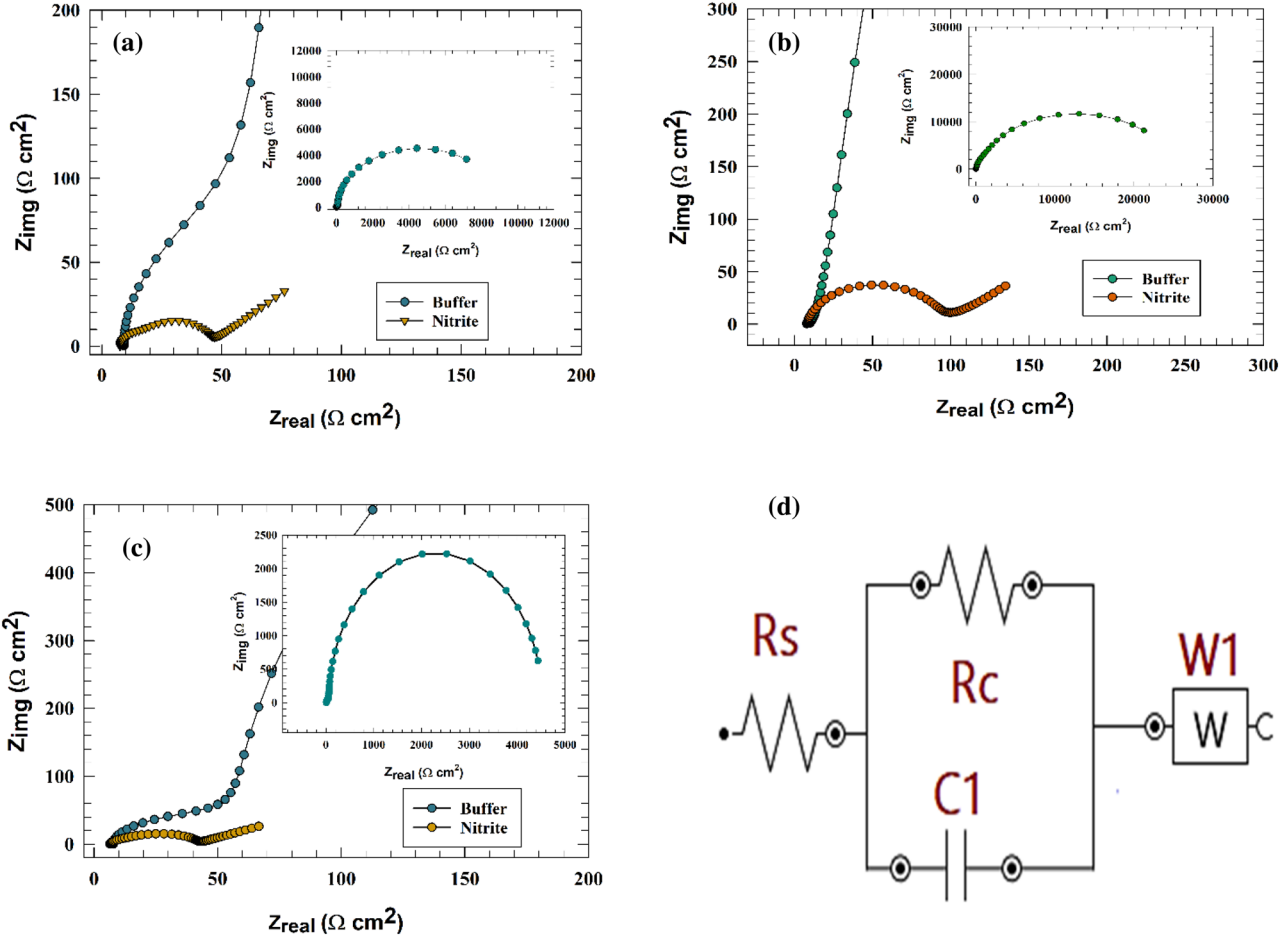
Nyquist plots of the modified GC/GO/Ag electrode at different pH values (**a**) 4, (**b**) 7, and (**c**) 10. (**d**) Representation of the fitting circuit of the modified electrode.

Electrochemical impedance was employed to ascertain the charge transfer resistance across various pH mediums. The Nyquist plot of a modified electrode (GC/GO/Ag) was analyzed in a solution containing 1.0 mM nitrite and 1.0 M NaOH at an AC constant potential of 0.9 V (vs Ag/AgCl), as illustrated in Fig. 8a–c. The observation of the single semi-circuit suggests that a one-charge transfer process should be considered along with the diffusion process. The equivalent fitting circuit that corresponds to the statement is presented in Fig. 8d. The Nyquist data can be fitted to the circuit of Rs attributed to solution resistance, R_c_ corresponding to charge transfer resistance, C for the capacitance of the electrode layers, and W (Warburg) element corresponding to the diffusion element. As shown in Fig. 8a, the Nyquist plot of the modified electrode in the presence and absence of nitrite at pH 4. In the inset, Fig. 8a shows the full scale of a modified electrode in a buffer. On the other hand, the neutral solution (pH 7), represented in Fig. 8b, showed higher resistance values than the acidic medium. Whereas, higher resistance values may reflect the lower activity of the electrode toward nitrite detection. Else, nitrite detection was investigated in an alkaline medium (pH 10) (see Fig. 8c). Thus, the lower impedance value for nitrite in an alkaline medium can be explained by higher efficiency for nitrite detection. Consequently, the EIS result could be used to confirm the results of CVs. The fitted data of the modified electrode (GC/GO/Ag) at different pH values are represented in Table 3.

**Table 3:** Fitting data for modified GC/GO@Ag electrode for different pH values.

pH	R_s_	R_c_	C_1_	W
	Ω cm^2^	Ω cm^2^	F	
4	8.4	63	0.0000796	0.000684
7	9.1	121	0.0000456	0.000544
10	6.3	53	0.0000945	0.000873

### Anti-interference ability

**Figure 9:**
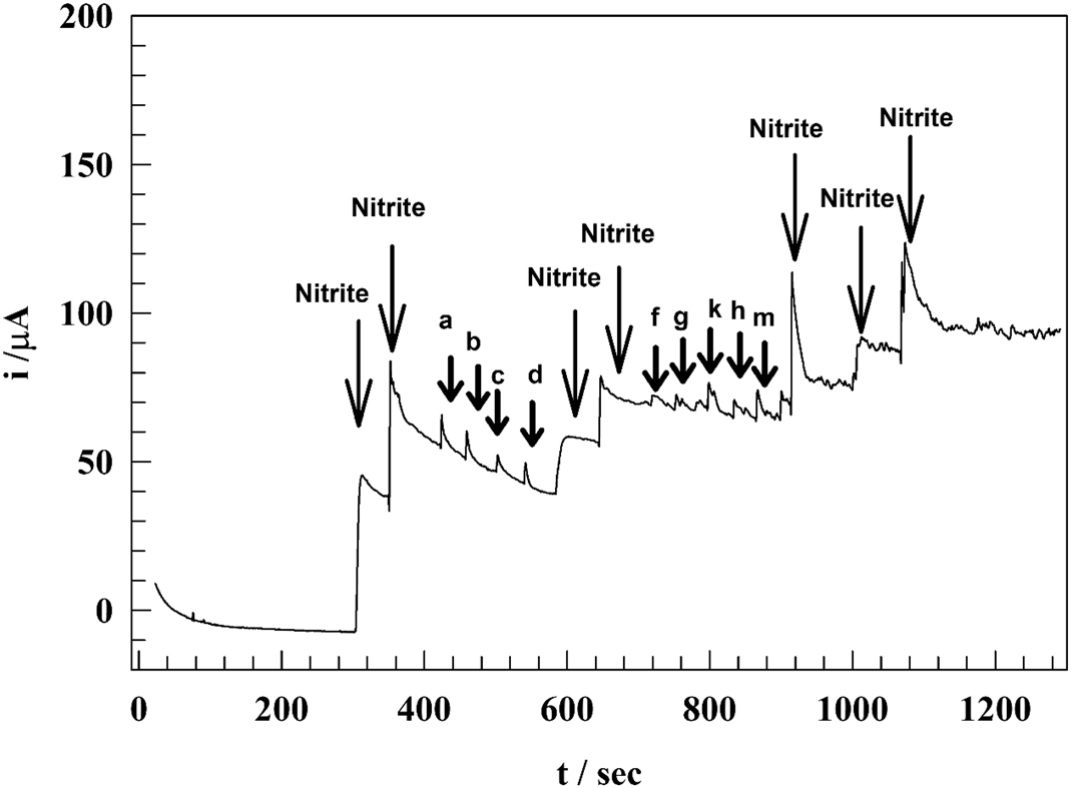
Chronoamperogram of modified GC/GO/Ag in the presence of different interfering species.

When determining nitrite in a wastewater sample, the GC/GO/Ag NPs electrode’s capacity to prevent interference is investigated using voltammetry. Common interfering ions (ZnSO_4_, AlCl_3_, NiSO_4_, and CuSO_4_) are utilized at high concentrations (100-fold) to examine their impact on the current response of nitrite determination used (as indicated in letter a–d). Additionally, significant levels (20-fold) of organic substances, such as uric acid and dopamine, are investigated for their ability to interfere (as indicated for letter f–m). However, in species used to measure nitrite in the presence of ions and chemicals, nitrite’s current response shows a slight modification (see Fig. 9). Results varied from 96.8 to 99.6% of the nominal value when ions were present and from 97.7 to 103.2% when organic compounds were present. The results refer to the GC/GO/AgNPs sensor’s ability to be selective and interference-resistant when measuring nitrite in the presence of metal ions and organic molecules.

### Nitrite detection in tape and wastewater

**Figure 10:**
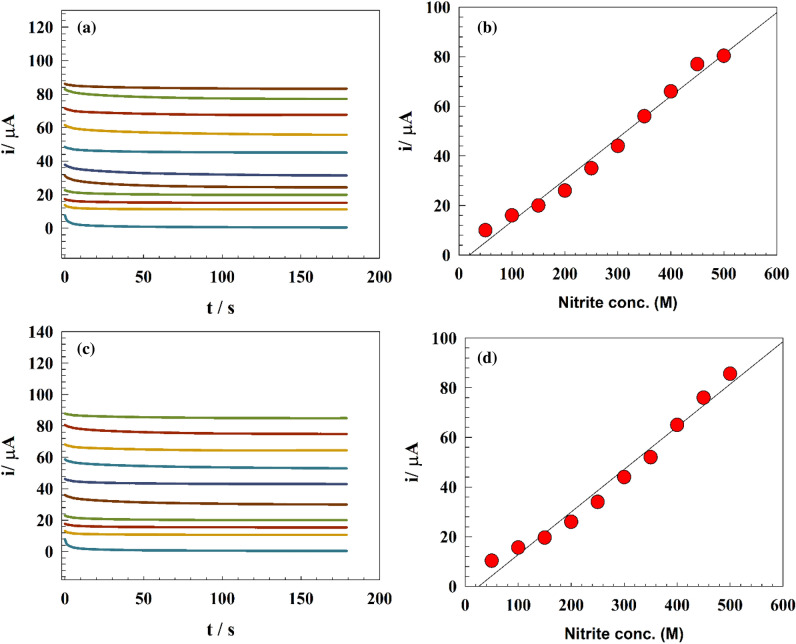
(**a**) Chronoamperograms of modified GC/GO/Ag electrode after spiking different nitrite concentrations in drinking water. (**b**) Calibration curve for drinking water samples. (**c**) Chronoamperograms of modified GC/GO/Ag electrodes after spiking different nitrite concentrations in wastewater. (**d**) Calibration curve for wastewater samples.

Electrochemical detection of nitrite is essential to determine the nitrite concentration in water samples. Therefore, real drinking water and wastewater were employed to characterize the electrode activity toward real-time application. The CA method was employed to determine nitrite concentration in tap water samples utilizing a modified GC/GO/Ag electrode. PBS adjusted the water’s real sample pH, and the pH was normalized to be equal to 7. Figure 10a,c show the chronoamperograms of the GC/GO/Ag at drinking and wastewater sample, respectively. The corresponding calibration curve of the drinking and wastewater samples are illustrated in Fig. 10b,d. Thus, both drinking and wastewater samples showed a linear range of detection in the range 25 to 500 µM. Additionally, the limit of detection is recorded as 0.16 and 0.157 µM for drinking and wastewater samples, respectively.

## Conclusion

To determine nitrite in actual water samples, a straightforward and sensitive electrochemical sensor is proposed based on modifying the GC electrode surface with two sequential layers of GO and Ag nanoparticles. When present in actual water samples with interfering ions and chemicals, the outstanding synergism of GO NPs and Ag nanoparticles and their unique properties as modifiers improve the nitrite electrochemical current response. The presence of graphene oxide in silver composite led to enhancement of the surface area and charge transfer process. The activity of GC/GO/Ag electrode showed high activity in both basic and acidic medium. Else, the high diffusion coefficient of the nitrite indicates the effective oxidation of nitrite on electrode surface. The electrode reported a low limit of detection for nitrite sensing at acidic, neutral, and basic medium. Additionally, the electrode GC/GO/Ag can detect nitrite effectively in tape and wastewater samples. Various organic and inorganic substances characterized the anti-interfering ability of electrodes.

## Data Availability

The datasets used and/or analysed during the current study are available from the corresponding author on reasonable request.
